# A Treatise on Operative Surgery, Comprising a Description of the Various Processes of the Art, Including All New Operations; Exhibiting the State of Surgical Science in Its Present Advanced Condition

**Published:** 1845-07

**Authors:** 


### Art. III.

*A Treatise on Operative Surgery, comprising a Description of the various Processes of the Art, including all New Operations; exhibiting the state of Surgical Science in its present advanced condition.* With Eighty Plates, containing 486 separate Illustrations. By Joseph Pancoast, M.D., Professor of General and Descriptive Anatomy in Jefferson Medical College, Philadelphia, &c. &c.—*Philadelphia,* 1844. 4to, pp. 380.

Such is part of the voluminous title-page of the latest surgical production of the American press that has reached us. It is of home growth, being the compilation of a native artist, and as such is entitled, on our part, to careful consideration; more especially as (we presume) it is intended to give an exposition of the art of operative surgery as it obtains in the Western hemisphere. We had observed the announcement of the work long before the volume came into our hands, and felt anxious for the opportunity of perusing an original treatise on the practice of surgery as followed by our American brethren. Here we apply the term "original" in contradistinction to the republication of the works of British authors on systematic surgery, with which the profession in America seem hitherto to have been contented. With Dr. Pancoast's name and professional reputation we were already familiar, and anticipated that we should find in his work a recompense for the careful perusal which we had resolved to bestow upon it. Our opinion on this subject we shall make known by and bye, but we must first carry our readers through the different sections into which the subject has been divided in the volume before us.

From an advertisement immediately preceding the table of contents, we perceive that the author has the desire that his work shall partake of the character of a "continuous whole," such as the comprehensive and elaborate works of Froriep of Berlin, and Bourgery of Paris, but in a less voluminous and expensive shape; and though he does consider it his duty to make some "brief observations on the therapeutical management of surgical affections," he seems anxious that it should be considered he has done so "without invalidating the claims of the work to be especially considered as a practical treatise on operative surgery." We cannot but perceive, too, that the pictorial illustrations are in the author's estimation not the least important feature in the volume. Hence it is evident that the principles of surgery,—that the common every-day duties of the surgeon, are to have but little consideration, and that our attention is to be directed almost solely to the mechanical department of the healing art.

The whole work is arranged into four sections: Part I being devoted to "Elementary and Minor Operations;" Part II, to "General Operations, or those practised with reference to one or more particular tissues;" Part III, to "Special Operations, or those which are practised upon complex organs in particular regions of the Body;" and Part IV, to "Plastic and Subcutaneous Operations."

In going through part First, in which are comprised, 1, "the division of parts with the bistoury and scissors; 2, division by ligature; 3, phlebotomy; 4, arteriotomy; 5, cauterization; 6, reunion by suture; 7, setons; 8, issues; 9, moxa; 10, acupuncturation; and 11, the means of arresting hemorrhage before, during, and after operation,"—a feeling came over us that we were looking only at what we had seen somewhere else—reading only what we had read before. We looked back to the "Advertisement" at the beginning, and being reassured, did not hesitate to proceed with part Second, which is devoted to "general operations or those practised with reference to one or more particular tissues." Here we found details of operations on veins and operations on arteries, set out with so much formality and precision—the old story of the "*process of Desault as modified by Delpech,*" the "*process of Lisfranc,*" "*ordinary process,*" "*process of the author,*" &c. &c.—that we grew sick at heart. We found that the same systematic parade which had often disgusted us with other compilers, was here also conspicuous. One man's plan was given with as much pomp as another's, the suggestion of the hero of the dissecting-room treated with the same consideration as the approved good work of the experienced practitioner, "*ligature of the ulnar near the termination of its upper third,*" "*ligature of the ulnar either at the middle or inferior third of the forearm,*" "*ligature of the ulnar below the pisiform bone;*" "*ligature of the anterior interosseal in the lower third of its course, (process of the author,)*" standing in the same page with "*ligature of the abdominal aorta,*"—the one process being, in the author's estimation, seemingly of as much importance as the other. From a party who volunteered a "Practical Treatise on Operative Surgery," in a quarto volume, and who had "the advantage of nine years' continuous service in one of the largest hospitals in North America," we naturally expected some practical observations of a somewhat valuable character on the various operations which have been performed, or been proposed on the arteries of the living body. We did find in the course of reading, that "the author" had accomplished some of those operations described; that he had tied the "femoral artery," (by which term he in reality means the superficial femoral,) four times, besides sundry other vessels, yet we felt not a little disappointed, as we concluded this section; we thought of the interest with which we had read Hodgson* in our younger days; how Harrison† had made the surgical anatomy of the arteries our delight; how Hargrave‡ imbued us with his own apparent zeal; we brought to mind the glowing description of Crampton, when he first saw the common iliac artery; how our heart beat as we read the account of Mott's operation on the innominata; how we almost feared to read further, as we perused Colles's account of his operation on the subclavian on the inside of the scaleni. We also called to mind what American surgeons had done. We remembered that Stevens had first tied the internal iliac; that Gibson, and after him Mott, had first secured the common iliac; that the latter had first tied the innominata; that Mussey and Mott had tied both carotids, a brief time only intervening between each operation,—in short, that American surgeons had done so much in this department, which was original. We were shocked to find the author proceeding in the same dull round of trite conventionalisms. "Place of election," "place of necessity," "process of Sedillot and Zang!" We hardly thought it in the nature of things that an American author could set about telling us that "Nuntiante Ippolito relates two cases in which the vertebral artery was tied at its origin with success," (though on what account we are not informed;) and yet omit even the mention of Dr. Post's name, an American surgeon who first successfully tied the subclavian artery!

And now having glanced from plate to plate, we felt that we were familiar with all their contents. The works of Velpeau, Liston, Lizars, Fergusson, Bourgery, rose in our 'mind's eye!'—BOURGERY! we rested not an instant until we could throw open his ponderous tomes. And, there, sure enough, we saw the Philadelphian originals, the "large number of accurate drawings," obtained during "nine years' continuous service," &c.! yea, verily, the whole "eighty plates," (with the exception of some two or three,) exactly, and to say truth, neatly, and accurately copied from Bourgery. Good, bad, and indifferent—all, or nearly all were there! We compared the first thirty or forty plates with those of Bourgery, and failed in finding the originals of some eight or twelve only of the "separate illustrations." Even some of these we traced to sources not acknowledged by our author. Although we have not taken the trouble to examine the whole, we are pretty sure we are not wrong in asserting that there are not fifty original sketches out of the much vaunted "four hundred and eighty-six separate illustrations."

But why, it may be asked, all this astonishment at a thing which is so common in America? The Americans have as much right to reprint medical as other books; and it would be surprising if they did not. Neither do we object to this work as being a "compilation." Any systematic treatise on surgery can be little else in the present day; it may be so done

* A Treatise on the Diseases of the Arteries and Veins, by Joseph Hodgson.

† The Surgical Anatomy of the Arteries, by Robert Harrison.

‡ A System of Operative Surgery, by Wm. Hargrave.

as to bear the stamp of as much originality as we might reasonably look for; and we expected such a work at least from Dr. Pancoast, more especially, when, on reading the "advertisement" prefixed to the volume, we received no hints to make us think otherwise. Here we are hardly given to expect so much as that the treatise is even *a compilation,* so carefully are the author's obligations to others kept out of sight. To be sure, he mentions the names of Froriep and Bourgery, as having preceded him in the attempt to collect the illustrations of surgical operations "into a continuous whole;" but who could for an instant imagine, from the following statement—the *strongest* one, in the Advertisement, bearing on this point —what is the real truth of the case?

"With these admirable treatises before him as a guide, and having at hand the greater portion of the surgical works, which have recently appeared in various languages, and with the advantage which nine years' continuous service in one of the largest hospitals of North America has given him, not only in comparing to a certain extent the value of the different methods, but in enabling him to obtain a large number of accurate drawings of operation which have been done by his own hand, the author has endeavoured to furnish a work that shall represent, so far as its limits will allow, the operative surgery of the day. In pursuance of this desire to portray the actual state of the science, many processes of operation have been given, for which the author cannot hold himself any farther responsible, than of having made of them a clear and impartial statement, drawn from the most authentic sources. The description of processes, too often given obscurely by their inventors, is confessedly difficult, and the author has not hesitated, when he believed he could thereby render their details more plain, to risk occasional repetition. The drawings, in almost every instance, have been represented in such a point of view, that the examiner may, in the stage of the process immediately shown, consider himself as the operator." (Advertisement.)

On reading this statement, who could help exclaiming, as we did— Here at last, is a treatise on operative surgery by an American surgeon, and just from such a party as we could have wished it? How heartily we accord with his opinion that such a treatise should be "thoroughly illustrated," and what may we not expect from a man who has had the treatises of Camper, Scarpa, Cooper, Hesselbach, Bell, Dupuytren, Froriep, Bourgery, together with "the greater portion of the surgical works which have recently appeared in various languages" before him, "as a guide;"— a man who has the additional "advantage which nine years continuous service in one of the largest hospitals in North America has given him, not only in comparing to a certain extent the value of the different methods, but in enabling him to obtain a larger number of accurate drawings of operations which have been done by his own hand." And the drawings, too, have been represented by this practical draughtsman and surgeon, "in such a point of view that the examiner may consider himself as the operator!"

Judge then of our disappointment when we found—what we have stated in the preceding page! The "orginals" among the plates were so far and few between, that it became almost a labour to find one out. We generally did so, however, by a certain indifference of execution, showing that another "hand" than that of M. Jacobs (the artist of Bourgery's work,) had been there. As a striking example of this we may refer to plate 55, in Pancoast, where we have a double illustration of the operation for excision of the mamma. One of them "taken at one of the operations of the author," contrasts most disadvantageously with the figure from Bourgery. It is professedly intended to show how "the chain of axillary glands enlarged and scirrhous," may be removed by an incision somewhat higher than that depicted by Bourgery, but it seems partly also to show that our author had actually done such an operation.

On turning to the letter-press of the work, we found the same or similar unacknowledged appropriation of M. Bourgery's, or some other surgeon's property. And, as we proceeded through the work, the conviction was irresistibly forced upon us, that the deception was seemingly as great in the one portion of the subject as in the other. The translations, no doubt, supplied favorable examples of Dr. Pancoast's powers in this way, for his numerous abbreviations did not in any material respect detract from the sense of the original, while they got rid of the prolixity of words so common with Bourgery, and most other French authors.

After this painful discovery, it now became a question with us whether we should proceed further. We had set ourselves to the task of reviewing an American author upon surgery, and found ourselves engaged in the perusal of a translation of a tolerably well-known French work, with which for the present we had no intention to meddle. Dr. Pancoast's mystification has been so much the more complete, as he has throughout carried on a kind of text of his own, which gives an air of originality sufficient to hood-wink those who may not be acquainted with Bourgery and the other authors on whom the "annexations" have been committed. Unquestionably, there is enough in this work of Dr. Pancoast's own, to show that he is a good practical surgeon; we nevertheless cannot perceive that his accumulated personal observations have been such as to warrant an essay of such apparent magnitude. His work is very defective on those very points in which we were especially interested; and we are greatly mistaken if his own countrymen will consider that he has done them justice.

Such a work as Bourgery's is one that we imagine may suffice for a generation; although the surgical part may not embrace all that we could wish. There is much in it that is mere matter of history, for few leading surgeons of the day are now interested in the principal processes which he details; they are historical records of what has already been done or recommended by those who have long since passed away. It cannot be overlooked, either, that Bourgery is more of a book-surgeon than one who performs the operations which he delineates; and it is vexatious to perceive that Dr. Pancoast, while he has detracted from what merit his prototype may have as a "continuous whole," has drawn none of those distinctions between the bad and what is in reality good and reasonably practicable, which might have been anticipated from one of his experience, but has reiterated a tissue of common-places and absurdities, quite intolerable to those in search of "the operative surgery of the day." In proof of this we may refer to the continuation of the "position" system with reference to holding a knife; eight different positions are detailed with all due gravity, each being illustrated with cuts; and these positions are further explained by as many more cuts showing how to hold scissors, and how to make certain incisions! The minute detail of the whole process is amusing on account of its grave formality, but as well might a boy, when he first goes to a boarding-school with his silver spoon and fork, have set before him a minute description of how he is to hold and use the same, as that our young surgeons should be taxed to learn these "positions," or rather to read the description of them. As a further proof we may notice some of our author's remarks about the position of the surgeon when performing an operation. He is about to describe an amputation in the leg, and thus he proceeds:

"*Position of the operator.* This is a point in regard to which there is a great diversity of opinion; some surgeons always placing themselves on the inner side of the limb, so that in dividing the bones the section of the fibula may be completed before that of the tibia, in order to guard the more surely against the splintering of the former. Others, believing the neat division of the soft parts a matter of greater importance, take a position always on the right side of the limb, so as to be able to grasp it with the left hand immediately above the place of operation. The latter I have found most convenient in practice, and a surgeon familiar with the use of the saw will have no difficulty even in operating on the right leg, of dropping the hand so as to divide the fibula before finishing the section of the larger bone of the limb. For the posture of the operator, the following minute directions have been given by Lisfranc: The right thigh flexed at a right angle with the pelvis, the leg bent upon the thigh, and the foot resting flat upon the floor; the left thigh flexed at an obtuse angle with the pelvis, the leg at an acute angle with the thigh, the tuberosity of the ischium supported upon the heel, and the point of the foot upon the ground, with the legs separated in order to give greater solidity and precision to his movements." (p. 165.)

We were curious to know where our author had got this minute description, but had not even to look into Lisfranc's works to satisfy ourselves, for on turning to the corresponding part in Bourgery (t. vi, p. 243,) we read the identical passage. High as Lisfranc stands in our estimation, we think it no detraction from his merit when we object to a repetition of such details. But as Dr. Pancoast and we do not agree on this point, and as possibly there will be some who think with him, we shall say no more regarding it than that, in our judgment, he would have evinced a more practical estimation of his subject by omitting such farcical minuteness.

In the first section of the work—elementary and minor operations—there is little original matter of an interesting character. Under the head "compound incisions" (*incisions composées* of Bourgery,) after the usual abbreviations with reference to T's, V's, &c., our author enlightens us thus, partly from Bourgery, and partly from his own stock—at least we have not leisure to look for another authority.

"*Elliptical and crescentic incisions.* The latter is only occasionally employed. The elliptical is in much more common use, and serves for the purpose of removing a portion of the integument, when it is redundant, as is often observed over the upper eyelid; or when it is deformed by cicatrices in parts like the neck or face exposed to view. It is employed for the removal of large tumours, as those of the testicle or mamma, in which the skin, either from its being too abundant or from its having suffered by the disease, requires also to be in part taken away. The lower limb of the ellipse in this incision should be made first, in order to avoid the embarrasment that arises from the flow of blood, when the upper has been previously formed. In many instances, and especially when the surgeon has not had sufficient practice to make him sure of his hand, it may be well to have the lines previously traced with ink or lunar caustic to ensure that the incision shall have its proper shape. Before using the knife the part must be made tense according to the directions given for the preceding operations. The *crescentic incision* is sometimes preferred to the elliptical for the removal of superficial parts, as the edges of the wound it leaves comes afterwards very nearly together. It is formed by two lines curved in the same direction, but belonging to circles of different diameter, that inclose between them a piece of skin thus 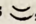 , which with the parts subjacent is to be removed." (p. 12.)

The almost obsolete rule regarding making the "lower limb" of the ellipse first, as also the recommendation to use caustic to mark the lines of incision are strange instructions for the practitioners of the 19th century, and how Dr. Pancoast can get his cresentic incisions to unite "very neatly" we cannot perceive. The cut we have given is a fac-simile of that in the text, and we imagine that if these lines were continued they would bear the mathematical relations to each other, that parallel lines do, that of never coming in contact. We presume that the Doctor meant to have given a cut of this sort 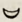 , whereby two portions of circles of different diameter are represented as coming in contact.

Dr. Pancoast affects originality in the operation of arteriotomy, which is described thus:

"1. *Process of the author*. A fold of skin about half an inch broad is to be raised above the vessel, and divided by a strait sharp-pointed bistoury, passed through its base in a direction somewhat oblique to the artery. If no other instrument be at hand, the section may be made with the thumb lancet. The lips of the wound are to be seperated with the thumb and fore finger of the left hand; the artery is to be laid bare with a few strokes of the point of the instrument, and punctured obliquely like a vein. The requisite amount of blood having been taken, the artery should be compressed with the finger below the wound and divided completely across. The retraction which follows usually stops the hemorrhage. The wound is then to be closed with two or three narrow adhesive strips, and secured with a double compress and roller. If the discharge is not immediately arrested a compress should be placed above as well as below the section. in order to prevent the return of blood by the anastomosing vessels. If the artery be large, a ligature for greater security may be placed upon it, or, which will usually suffice to stop the blood, the wound may be closed with a stout hare-lip suture.

And by way of contrast he gives us what he is pleased to call the "*usual process.*"

"2. *Usual process*. The position of the artery being marked with ink, and the skin made tense above it with the thumb and index finger of the left hand, the artery is divided completely across with the convex-pointed scalpel, which should be pressed downwards directly upon it with the fore finger upon its back till it meets the bone, and then drawn slightly towards the operator." (p. 19.)

We think we can answer for our countrymen, this is not the *usual process* followed in this part of the world, and if it be that used in America the sooner it is forgotten the better: our author would have done well to have omitted the description altogether.

On the subject of cauterization we have a good account of the various kinds of potential cauteries, more especially of the different forms in which the chloride of zinc may be used, but no striking cases are given of its successful application.

In the rules for the application of sutures, our author states that "it usually suffices to pass the needle merely through the skin and subcutaneous cellular tissue; but in cases of deep cuts involving the muscles, or in wounds following resection or amputation, they may also pass with advantage through a portion of divided muscle." We doubt if the generality of our surgeons would agree in the latter part of this doctrine, and for ourselves, after considerable experience in resection and amputation, we should deem the practice of passing stitches through muscles objectionable on many grounds.

The first section of the work concludes with some observations on the means of arresting hemorrhage during and after operations. After enumerating and describing the methods resorted to by Amussat, Fricke, and others, it is sensibly remarked that "several of the various processes above detailed may be found occasionally useful in practice; but the surgeon who would wish to leave his patient with the nearly positive certainty that he will not be troubled with secondary hemorrhage, should tie the vessels," an opinion in which we are glad to say we most heartily concur.

Part Second, opens with "operations upon the veins." Transfusion of blood is first noticed as in Bourgery's arrangement, and then with some little changes here and there, we have the operations on varicose veins dished up *à la Bourgery*. We are pleased however to perceive that the author gives us a little of his own experience in such cases, and for the sake of doing him full justice, we shall quote as follows:

"*Compression over a pin or needle.* (1*st Process of Davat.*) Raise the vein in a fold of skin, through the base of which and below the vein a pin or needle is to be passed transversely. Around this needle is to be wound a harelip suture, sufficiently tight to keep the anterior and posterior surfaces of the vein in close contact. Several pins, from four to ten or twelve, should be employed at little distances from each other, upon the main trunk and its principal branches, so as to cut off effectually the route of the blood through the superficial veins, and cause it to return by the deep-seated. Velpeau prefers to surround the two ends of the pin merely with the thread in vertical turns, rather than in the form of a figure ∞, as it is less disposed to cause ulceration of the skin. An elliptical wrapping of the pin, however, as shown at fig. 4, is decidedly preferable to either.

"2*d Process of Davat.* After the introduction of one pin, as above described, a second is to be entered a little lower, perpendicularly *through* the skin and both surfaces of the vein; it is to be carried in the direction of the vein under the first pin, and brought out on the opposite side, piercing a second time the two surfaces of the vein and that of the skin. The two pins are at right angles with one another, and are each to be wound with the hare-lip suture. In my own practice, the first process has answered best. When the vein, as for instance the saphena on the thigh, is covered by a layer of superficial fascia, it is difficult to raise it up so as to pass the second pin readily in the prescribed longitudinal direction. Its effect also has appeared to be rather injurious than otherwise in producing two transverse folds of the vein, which keep the sides from coming so well in contact as when the single pin or needle is passed across and covered with a compress and bandage. From the sixth to the tenth day the obliteration will be usually found complete, and the pins may be removed. I have several times employed two or three separate pins in this way, upon the saphena along the inner surface of the thigh, when the enlargement of the vessels had extended from the leg upwards upon this region; while others were introduced concurrently upon the vessels of the leg. In no instance have I failed by this method to produce a cure, or very marked amelioration. A bandage wound tightly on the extremity from the groin downwards, and perfect rest in the horizontal position, were the means employed to guard against the risk of the supervention of phlebitis, which, as reported by Velpeau, Lallemand, and Serres, has in some instances been attended by fatal consequences." (pp. 37-8.)

"Pour prévenir l'invasion des phlébitis mortelles, comme il dit être arrivé à MM. Velpeau, Lallemand, et Serres," as Bourgery has it.

We believe that the practice of meddling with the saphena on the thigh has not been often attempted in this country. We have ourselves tried it occasionally, though at the risk of being considered rash, and have observed the same immunity from danger, as has resulted in Dr. Pancoast's hands. Interfering with veins has been a bugbear with English surgeons for the last forty years or more, and we think it strange after the vast amount of modern experience, that there should still remain such a dread of touching them. We have seen varicose veins cut, torn, burnt with the potential and actual cautery, and tied in almost all methods, as innocuously as in similar proceedings with other tissues. We should have been pleased if Dr. Pancoast had given us some numbers, or referred to the practice of other American surgeons, to show to what extent this modern method of treatment has been followed out in that part of the world.

Our author being a teacher of anatomy, luxuriates on the "operations upon the arteries;" like most others of the same class, he dwells with as much minuteness upon the ligature of the fibular artery, as upon any of the iliacs; on ligature of the occipital artery as upon that of the carotid. He seems almost to go beyond Bourgery in telling what things may be done. We cannot resist a quotation here, were it only for the edification of some of our prosectors:

"*Remarks.* The ligature of this vessel [occipital artery,] has not yet, I believe, been made upon the living subject. The position of the artery is such that in cases of wounds involving it, it may either be secured at the place of injury or compressed against the bone. Circumstances, however, may possibly arise,—such as aneurism, or a tendency to erysipelas presenting an obstacle to compression,—that may render the ligature necessary. A wound of the vessel near its origin, in consequence of the depth at which it is placed, and the difficulty of ascertaining precisely the trunk from which the hemorrhage arises, must be met by ligature of the external or primitive carotid.

"*Operation.* The scalp having been shaved behind the ear, an incision is made through the skin an inch and a half to two inches long, beginning it at the posterior border of the sterno-cleido-mastoid, about a half inch behind and a little below the point of the mastoid process, and carrying it obliquely backward and upward in the direction of the superior curved line of the occipital bone. The aponeurosis of the above muscle is next divided, and the splenius exposed just below the line of insertion. The splenius is next to be divided the whole length of the wound, either by incision from above downwards with the knife, or on the groove of the director. The artery, which may now be felt pulsating, is to be isolated and tied. Particular care should be taken, as observed by M. Manec, not to open either of the accompanying veins, as from their connexion with the lateral sinuses of the brain through the mastoid foramen, they would bleed very freely." (pp. 50-1.)

Then follows, in the same grave strain, a description of the surgical anatomy of the posterior auris, with "remarks" and "operations," &c., all formally displayed.

In the remarks on deligation of the subclavian "within the scaleni," we observe an error of a serious kind which however, we are willing to impute to an oversight on Dr. Pancoast's part; "on the left side," he states "it has been but once tied, (by Mr. Colles of Dublin—the patient died on the ninth day,) in this portion of the vessel, and the complicated surgical relations which it has in that region, will serve to show that the operation, though not wholly impracticable, must be hazardous in the extreme." We well remember Mr. Colles's description of his operation on the right side, a proceeding which has been repeated on various occasions since, but must admit our ignorance regarding any such proceeding on the left; it appears to us that our author is altogether confused on this point.

Dr. Pancoast seems to have had considerable experience with regard to wounds of the humeral artery inflicted during venesection, and we have great pleasure in quoting from this part of his narrative:

"As soon as the injury of the artery by venesection or other means is detected, it is incontestably the surest course at once to recur to the ligature of the vessel, in order to prevent either of the consequences that may follow—the common form of false aneurism, varicose aneurism, or that to which I have limited the term of aneurismal varix. Two methods of proceeding are then open to the practitioner—to incise the parts at the bend of the arm, and to tie the artery above and below the place of puncture; or follow the method of Hunter, and tie it where it is more readily exposed in its course along the biceps muscle. If the operation is done *shortly after the occurrence of the injury*, the former method is not ordinarily the best, inasmuch as it is desirable to avoid an incision at the elbow, in consequence of the deeper covering of the artery, its complex relation with the veins of that region, and its obscuration from the extravasation of the blood which to more or less extent takes place. The method of Hunter is a more simple process, and if soon applied is equally successful; to which compression may if necessary be added at the bend of the arm; for it has been fully proved by experience, that the anastomosing vessels will not dilate so as to restore the circulation in the wounded trunk till sufficient time has been allowed for the healing of the puncture made in it by the lancet. A great accumulation of effused blood at the bend of the arm, pressing on the origin of the recurrent radial and ulnar arteries, might, however, as a case of exception, render it better to cut down, turn out the clot, and tie the brachial above and below the place at which it is wounded.

"The principles involved in the Hunterian operation, of tying the artery at a remote distance from the tumour, are not so binding here, where we have to deal with a sound vessel accidentally injured. A distant ligature, though it may answer if applied immediately after the injury, is not to be relied on in case much time has elapsed since the occurrence of the injury, if a large aneurismal tumour has been formed, or if compression has for some time been made from without; for from all these causes the anastomosing branches become enlarged, and the blood will find its way into the trunk at the elbow, both by the inferior arteries of the joint and the superior branch called the anastomotica magna. For these reasons I prefer always to tie the trunk an inch to an inch and a half above the joint and below the origin of the anastomotica. This simple operation has succeeded perfectly in my hands in four cases, which were respectively of four, five, eight, and nine weeks, standing, in each of which, tumours of considerable size had already formed. In another of nine weeks' standing, a case of proper aneurismal varix, upon which firm pressure had been steadily kept up, so as to cause great enlargement of the profunda minor, the pulsation of the veins, though not entirely removed by the ligature of the brachial, was and still remains considerably reduced by the operation, so that the arm has been restored to very nearly its

LIBRARY OF THE

Orleans Parish Medical Society former degree of usefulness. A circumstance connected with the operation in this case is worth noting;—pressure upon the brachial through the integuments above the elbow stopped all pulsation in the artery and veins below, the profunda minor, which was afterwards found greatly dilated, being at the same time in the line of compression. But after the ligature of the brachial, the profunda served to keep up some pulsation in the vein, through its anastomosis with the vessels below the joint." (p. 61.)

We wish that Dr. Pancoast had shown his acquaintance with the very successful practice of the late Mr. Tyrrell, by pressure in such wounds; nevertheless we cannot read our author here without expressing regret that he had not written the whole of his book in the same practical strain and from his own stores. We feel that we should have had a very different task to perform than that on which we are at present engaged. Notwithstanding our objections to the minute details in this section, we feel bound to say that here the author considerably excels Bourgery. His historical details though far from being complete, are more perfect than those of the Parisian author. We cannot, however, forgive the neglect of his own countrymen in this interesting department. In a work where so much has been quoted both openly and furtively, we should have liked to have seen the manly accounts given by Mott, of his first operations on the innominata and the common iliac. He does state that "the honour of having first performed this most serious operation, is due to Professor Mott, of the University of the city of New York," but on the same page in which he describes the "process of Mott," we have the "process of King," chiefly, we suppose, because Bourgery has done the same. Why not rather have given "the process" of some other party who has accomplished the operation on the living body? This want of discrimination between great and little authors, as between great and little processes, displeases us greatly in a work professing to be of a practical character. King, we suppose, used to show his operation in the dissecting-rooms in Paris; hence the reason, probably, that he is given as an authority by Bourgery; but that an American author should follow up Mott's name with that of King, awarding almost as much notice to the one as to the other, does indeed not a little surprise us; especially when we couple this with other omissions, or, it may be commissions, of a like kind on his part. We hope that some friendly American will enter the lists in favour of Mott, Post, Gibson, and others. We should have much pleasure in doing so ourselves; but grateful though the duty would be, we should prefer to see our Philadelphian author made to run the gauntlet by one of his own countrymen,

Further on we are glad to perceive that Dr. Pancoast gives his countrymen Drs. Barton, Rogers, and Gibson, all the merit which they deserve for their bold and ingenious operations in cases of anchylosis, and as these proceedings are not familiarly known in this country, our readers will doubtless feel interested in the details.

"*Formation of an artificial joint.* This method, for which we are indebted to the ingenuity of Dr. John Rhea Barton, of this city, has been applied as yet but to the anchylosis of a single articulation—that of the hip-joint. It has, however, been suggested by this skilful surgeon, that it might likewise be found applicable to similar affections of the lower jaw, knee, elbow, fingers and toes, when the muscles of these respective articulations remain uninjured. The method consists in the uncovering of the bone at or near the diseased point, dividing it across with the saw, and subsequently moving the lower portion from time to time upon the upper, to prevent a solid reunion of the divided parts. By this mode of proceeding, there is the same disposition of parts for the formation of a false joint, as we often find producing that result in fractures where the bones are not kept sufficiently at rest. Under such circumstances, the two opposing surfaces of bone may be expected to unite by flexible ligamentous matter, or become smooth and polished by the friction; the lower fragment, in the latter case, rounding itself into the form of a head; and the other hollowing itself more or less into the shape of a cup, in which the former plays; the periosteum and surrounding cellular tissue becoming condensed and thickened, so as to perform the office of a fibrous capsule, and the muscles modified to a certain extent, to accommodate themselves to the new articulation.

"*For anchylosis of the hip.* The ingenious idea of remedying this deformity by the establishment of an artificial joint, was first practised by Dr. Barton in 1826. A similar operation was repeated four years subsequently by Dr. J. Kearny Rogers, of New York; the two constitutiug the only instances in which it has yet been attempted on the living subject. The patient of Dr Barton was a young man twenty-one years of age, in whom the thigh was held immovably bent at a right angle with the pelvis, and the foot turned in rotation inwards. A crucial incision was made over the projecting portion of the trochanter major, the vertical division of which was seven inches in length, and the transverse five. The four laminæ thus formed were dissected and turned back, and the fascia freely opened. The muscular fibres were then detached from over the trochanter by turning the scalpel sideways, so as to allow the two index fingers to be passed freely round the neck of the femur, till they met on the opposite side. With a strong straight saw the bone was then nearly divided through the upper part of the great trochanter and part of the neck of the bone. The operation lasted but seven minutes, and no artery was opened that required to be tied. The limb was then drawn to its proper position, when the undivided portion of the bone separated with a snap. The wound was closed with a few points of suture, and the extremity secured in the fracture apparatus of Desault.

CHARITY HOSPITAL, MEDICAL LIBRARY.

"On the twentieth day after the operation the inflammatory symptoms had in a great measure subsided; some slight passive movements were then made with the limb, in directions natural to the healthy joint, which were cautiously repeated from time to time. By the sixtieth day the wound was completely healed; the patient was able to stand erect with the aid of crutches, and could advance his limb exclusively by muscular exertion. At the end of four months he was able to walk without apparent lameness, and all the movements of the limb were executed without pain. The foot could be carried twenty-two inches forward, twenty-six backward, and twenty outwards, and could be rotated inwards to the extent of six. The patient enjoyed the use of his artificial joint for a period of six years, at the end of which period, from causes attributable to intemperance and repeated falls upon the hip, the new joint became permanently anchylosed.

The operation of Dr. Rogers was equally successful, and his patient left the hospital at the end of four months, apparently with a perfect use of the new joint, as he could walk with ease by the assistance of a cane. Of the ultimate result in this case—whether or not the new joint in the end became anchylosed, as in the case of Dr. Barton, the profession has not been informed. In consequence of the shortening of the limb of the opposite side from fracture, Dr. Rogers, instead of making a simple section, removed a wedge-shaped portion of the bone, in order to render the relative length of the two limbs more equal.

"In place of dividing the bone after section of the soft parts, as above described it has been proposed, by M. Louvrier, to produce directly by mechanical means a fracture of the neck of the thigh-bone, a measure which he believes less dangerous than the former, and affording equal facilities for the formation of a false joint. But provided it were possible to succeed in fracturing the bone at the desired point, there would be such danger by this method of doing violence to the surrounding parts, that it can offer no probable advantages to cause it to be compared to the neat and methodical section of the bone according to the method of Dr. Barton. It would be rather more easy to divide the femur below the trochanter, but by this method an all-important object would be lost—that of obtaining a new and solid articulation upon the pelvic bones, so as to reestablish the functions of the limb with the least possible shortening." (p. 89.)

This is followed by a description of Dr. Barton's bold and original "operation for straightening a bent and anchylosed knee-joint," which we noticed at the time in our Sixth Volume, p. 254. A similar operation, and with the like success, has been performed by Professor Gibson of Philadelphia.

We think the author has erred in judgment in giving the details of the disgusting process of Louvrier for straightening the knee-joint. The description of the "stretching apparatus" and the effects which are produced with it makes us shudder as if we were in the torture-room of the Inquisition. We should have been quite satisfied had the subject been merely alluded to; at the same time it would have been gratifying had Dr. Pancoast adverted to Dieffenbach's proceedings in such cases, and also favoured us with his views thereon. In our opinion it speaks well for our author's sense, when he thus remarks upon Louvrier's practice, "the consequences of these attempts have not been such as to sanction the adoption, especially as regards the larger joints, of a highly dangerous experimental operation, for a mere deformity, which does not in itself compromise life."

The successful issue of the operations performed by Barton and his followers are sufficient to arrest the attention of the surgeon. We are not aware that any similar operations have been performed in this country, nor do we think them of very general application; yet cases may from time to time occur wherein we might with advantage consult this leaf in American surgery, and we perceive that a case is alluded to wherein a great deformity of the leg resulting from a badly set fracture, was successfully treated by our author's colleague Professor Mütter, by cutting out a wedge-shaped portion of the callus.

It is only justice that we should speak in high terms of our author's observations on trepanning the bones of the cranium. Our limits will not permit us to quote them, but we have much pleasure in stating that they are highly to his credit, and betoken a well-stored mind. Dr. Pancoast has evidently studied the subject deeply, and this observation again leads us to express regret that he should have compromised himself so greatly by his servile imitations in so many other things.

A large space is devoted to the consideration of resection of bones, and at the commencement (p. 108), we are startled with the assertion that the chief source of danger after such operations is "the development of tetanus." We have no proof given that such a result is of common occurrence in America, certainly with us it is a rare event. Resection of the jaws, more especially the upper one, is dwelt upon at great length, and here as elsewhere the illustrations are chiefly drawn from Bourgery.

Excision of the elbow-joint, seems as yet a novelty with the Americans. We give the following account of an operation by the author, as at once very creditable to him as a surgeon and a good specimen of his own style when he chooses to speak for himself.

"The patient was placed with his face downwards on a bed, over the side of which his arm was extended and supported by an assistant. Another assistant steadied the shoulder, and restrained the movements of the patient. A bistoury was now entered perpendicularly into the joint, on a level with the top of the olecranon, with its back almost in contact with the ulnar nerve, as directed by Syme, and the integuments, triceps tendon, and capsule, divided with a sawing motion completely across to the external tuberosity of the arm. From either end of this transverse incision, the integuments were divided through to the bone upwards as well as downwards for an inch and a half along the opposite margins of the arm, so as to give the wound the shape of the letter H, and form the two square longitudinal flaps of Moreau. The ascending incision, on the ulnar side of the arm, was inclined at its *commencement* a little towards the radius, for the purpose of more surely avoiding the course of the ulnar nerve. The flaps were dissected from the surface of the bone, and reflected upwards. The upper one was so loosened by suppuration from the end of the humerus, as to be readily stripped off. Its reflection upwards was more difficult in consequence of the great effusion of ossific matter in the cellular tissue on the side next the bone. The olecranon process was then sawed off at its base, in a direction slightly sloping towards the joint. The surfaces of the bones forming the joint were now well exposed to view; the ends of the humerus and ulna were found extensively affected with caries, and the synovial membrane of the interior of the joint, as well as that of the lesser sigmoid cavity, was soft and pulpy. The caries had not, however, extended beyond the articular epiphysis of the bone, though each bone at a considerable distance from the joint was thickened, and rough, and reddened by granulations in the process of formation. The ligamentous structures on the sides of the articulation were now cut through with the knife; care being taken in dividing the internal lateral ligament to loosen previously the ulnar nerve from its bed, and press it inwards with the left thumb, while the bistoury was introduced between it and the ligament with which it lies in contact.

"The arm was then bent and the radius twisted forward, so as to expose completely the external condyle. This was divided with a Barton's saw from a point just below the tuberosity nearly into the sigmoid fossa. The internal condyle was sawed in a similar manner, the forearm being twisted in the opposite direction, and the ulnar nerve pressed off with the thumb. The division of the bone was then completed with a pair of strong cutting pliers, partly by splitting and partly by cutting. Subsequent experience has, however, convinced me that a thin wedge shaped chisel, forced into the groove of a saw with the tap of a mallet, answers under such circumstances still better than the cutting forceps. The articular face of the bone thus separated from the shaft through the sigmoid fossa, was twisted off with a large pair of curved tooth forceps, and detached with the point of the knife. The ulna was now made to project backwards, and the soft parts separated from it on either side with the bistoury, for the space of three quarters of an inch; the knife being carried on with the edge in contact with the inner side of the bone, so as to avoid the nerve which runs parallel with its sloping surface. The carious head of the ulna, which was soft and filled with fatty matter, was then detached with the saw and chisel, and its whole articular face with the point of the coronoid process twisted away with the forceps. The base of the coronoid process was not removed, as this was covered with the insertion of the brachialis anticus, and forms no part of the joint. The head of the radius, on which the cartilage was softened, was pushed up so as to project from the orbicular ligament, and snipped off with the cutting pliers. All the pulpy portions of synovial membrane, including that of the two sigmoid fossæ, were removed.

"The forearm was now placed in a middle position between pronation and supination; but to do this it was necessary to divide the orbicular ligament of the radius, which resisted the movement. The cavity of the wound was sponged clean of blood, two small arteries were tied, and the flaps closed with six sutures passed merely through the integuments. The elbow was but slightly bent, in order to favour for the first five days union by first intention in the divided integuments. Simple dressings were applied, and a patent felt elbow-splint well padded secured round the joint with a figure of 8 bandage. The arm in addition was fastened to a pillow, and rested upon an inclined plane. About eight ounces of blood were lost during the operation.

"The patient was placed under the free use of a solution of morphia in camphor water, and directed to keep the wound well wetted with a cold astringent lotion. The wound united nearly throughout by first intention, and notwithstanding a slight attack of pneumonia, which came on after the operation, he was sufficiently well on the ninth day to leave for his residence at a distance of about twenty miles from the city. Passive motion was directed to be kept up for a considerable period in the joint; and though the injunction was but imperfectly complied with, the patient has preserved a strong and useful arm, with free flexion and extension at the elbow." (p. 123.)

Our readers will perceive that this is nearly the same operation as has been so frequently and successfully performed by Mr. Syme of Edinburgh, and we hope that it will be appreciated by the Americans, as it is by all good surgeons in this country. Mr. Syme's operation, we believe, differs from that of Moreau and most others in this, that he takes away the smallest possible amount of bone,—that is, the diseased portion only, leaving the portions thickened with the new deposits alone.

The resections of bones in every region of the body are dwelt upon with a minuteness that should gratify the most fastidious anatomist, and Bourgery's work is of course largely drawn upon. We cannot, however, perceive anything likely to interest our countrymen, unless it be this:

"Resection of the first Metatarso-Phalangeal Articulation. *Process of the author.* In 1836, I removed at the Philadelphia hospital the entire metatarso-phalangeal joint of the first toe, preserving two thirds of the first and the whole of the second phalanx. The case was one of caries, caused by a spike nail run through the joint. The whole structure of the articulation was swollen and thickened, and two fistulous openings existed low down on the sides of the foot. I made a semicircular incision, which traversed these openings, and dissected the flap, the base of which was towards the heel, so as to turn it backwards upon the foot. This exposed completely the inner surface of the joint, and about half the length of the metatarsal bone. The joint was next opened, the metatarsal bone isolated from the tendon and the surrounding parts, and divided across near its middle with the metacarpal saw. On the removal of the fragment, the end of the phalanx was found carious: this was pushed out through the wound, and a portion a quarter of an inch long removed with the saw. The interior structure of the adjoining part of the phalanx, which was soft and spongy, was scooped out with the end of the scalpel. The ends of the divided bones were then put in contact, and the flap brought down and secured with adhesive straps and a retaining bandage. Some suppurative discharge continued for three weeks at the posterior angle of the wound; but it ultimately healed up well. Solid union took place between the divided bones, and the patient preserved his toe, which was found after the cure about three quarters of an inch shorter than the other. The only difficulty encountered in the after-treatment, was the tendency of the extensor muscle to elevate the point of the toe. Should I again have occasion to excise this joint, I would prefer to divide this tendon, in case I approximated the bones, inasmuch as the necessity for its use would be greatly diminished afterwards; the middle phalangeal joint, in regard to position and office, supplying the place of the one excised; and there would be reason to expect that the reunion of the divided tendon would be sufficiently perfect to prevent (in conjunction with the dressing) the flexor muscle from drawing the point downwards. M. Petriquin reports a similar operation done by Professor Regnoli, of Pisa, in the case of a girl 20 years of age, and which he saw in the progress of cure. By this mode of operation we preserve well the shape of the foot." (p. 132.)

It has often been our lot to remove the great toe and metatarsal bone, in cases similar to the above, and we confess that the idea of resection never entered our mind, yet we shall think of it in future. In so far as our knowledge extends we believe Dr. Pancoast to be original in this process, and feel obliged to him for its introduction. We regret that no notice is taken of the effect of anchylosis in this part, but presume, since nothing is said to the contrary, that it produced no inconvenience during progression.

Our author commences the subject of amputation with some judicious observations in regard to the propriety of attempting to save a limb instead of seeking this last resource. He at the same time gives caution as to protracted delay.

"It is not," he states, "perhaps saying too much, when I aver, from the frequent opportunities which I have had of witnessing their performance, and the fair share that has fallen to my own lot, that from a combination of erroneous judgment, and a mistaken notion of humanity, the performance of these operations is frequently deferred until their chances of success when practised have been considerably compromised."

We have ourselves seen amputation performed, when in our opinion the process might have been dispensed with or delayed in hopes of a favorable issue otherwise, but feel satisfied that we have seen more gross errors by procrastination. In the alarm and bustle induced by some fearful accident, the surgeon may, on the emergency, commit a blunder which he would not during his calmer moments, but when a case has assumed a chronic character, and there is full time to deliberate, we hold him unpardonable who sees from day to day, that the disease still gains way over the constitution, and yet does not interfere, even though it be with this last of all measures calculated to save life. We hold it as the result, and as the test of experience, that when the case is left entirely to the judgment of the surgeon, he should at the proper time say that amputation should be done. It shows little in the practitioner's favour that he waits until the patient or the friends insist on an operation. At a certain period even a non-professional person can form a tolerably shrewd opinion as to the necessity of such a proceeding, and the surgeon is scarcely to be justified who shall at this juncture declare that it is now too late; that the favorable opportunity has passed by.

It is difficult to make out which operation our author himself prefers. We have "circular method," "flap method," "oblique or oval method," "mixed method," "1st process of Lisfranc," "2d, do. do.," "process of Cornuau," "process of Baudens," "do. of Dupuytren," "of Grosbois and Dupuytren," arranged in the due Bourgery style, but a "process of the author" is of rare occurrence. Almost the entire of this portion of the work seems a counterpart of Bourgery, with here and there a dash of novelty from some other source, and occasionally a little of the author's own material. From this last we are glad to be able to extract an account of his method of performing amputation at the knee-joint:

"*Process of the author. Three cutaneous flaps.* The patient is to be placed upon the abdomen. The leg, flexed at a right angle with the thigh, is held by an assistant. The surgeon, placing the thumb and forefinger upon the condyles of the tibia at the opposite sides of the leg, makes with a common scapel on the front of the upper part of the leg, a semilunar incision which extends as far as three inches below the tubercle of the tibia, one extremity resting on either side an inch below the joint. The flap of skin is now to be rapidly dissected towards the joint. The leg is then to be extended and made horizontal. The point of the knife is next to be entered through the skin at the middle of the back part of the leg, an inch and a half to two inches below the fossa of the popliteal space, and carried vertically downward for the space of three inches. From the lower end of this, the knife is to be continued round on one side to strike the line of the first or anterior incision, so as to mark out a second flap, convex downwards, and extending a little lower than that of the one in front. The lower end of the vertical cut is then united by a similar convex sweep of the knife to the other margin of the front incision, so as to form a third flap. The two posterior flaps are next to be dissected from the fascia up to their base. The leg is now to be again flexed, and from the general loosening of the flaps already made, the insertion of the ligamentum patellæ upon the tibia will be exposed. This is to be divided across and the joint opened upon the front and sides so as to leave the semilunar cartilage on the head of the tibia; the crucial ligaments, as they become subsequently useful as a nidus for granulations, are to be divided at their connexion with the latter bone, and the posterior ligament lastly cut. The leg, which is now loose, is to be twisted on the thigh. An assistant grasps the popliteal artery with the thumb and finger, and the surgeon divides below at one stroke with the knife the remaining parts, consisting mainly of the two heads of the gastrocnemius, some of the hamstring tendons not previously cut, and the popliteal vessels and nerves. The patella is to be left in its position. The whole operation may be done with the scapel; the femoral artery should be compressed with the tourniquet." (p. 169.)

A sketch is given of the stump, and we may notice here, in passing, that the figures on the same page are among the few not taken from Bourgery. Their execution is evidently inferior to the copies from the Parisian artist.

The drawings are exceedingly useful to this part of the work, and we imagine that no apology would be admitted for a pupil of Dr. Pancoast's, were he to plead ignorance regarding any of the numerous processes which it seems his master's pleasure to teach. For our part, however, we should be lenient with the youth even if he did so, for our own brains have been somewhat muddled with processes "circular," "flap," "oval," and "mixed," with the usual quantity of dates and proper names, often of parties of whose "whereabouts" most people are in total ignorance.

We have now carried our readers over the first half of the volume, and fancy that we may be excused from minute consideration of the two remaining sections. With the exception of several illustrations of plastic operations, including some clever examples of nose patching, in which business our author seems to excel, nearly all the drawings are copied from Bourgery, and the letter-press is a sort of free translation from the same work, hashed up with Dr. Pancoast's additions. American surgeons seem to have done a good deal in plastics, and this part of Dr. Pancoast's work is interesting; but as we have devoted many of the pages of our preceding Number to this subject, we will not dwell upon it here.

The various processes for the radical cure of hernia are carefully illustrated, (from Bourgery of course;) but we are happy in earnestly calling the attention of our own surgeons to the following practice of the author himself, which we have much satisfaction in quoting:

"*By injection.* This process, as employed by the author, is as follows. The contents of the hernia must be completely returned into the cavity of the abdomen, for the process is only appropriate to cases of reducible hernia, and those which are not of large size. The apparatus required is a minute trocar and canula, a small graduated syringe, capable of containing a drachm of fluid, well-fitted to the end of the canula, and a good-fitting truss for the purpose of making compression. The patient is to be placed on his back; the viscera are then to be reduced, and the truss applied over the external ring for the purpose of keeping them up, as well as to prevent the possibility of the small quantity of fluid thrown in from getting into the cavity of the abdomen. The surgeon then presses with the finger at the external ring so as to displace the cord inwards and bring the pulpy end of the finger on the spine of the pubis. At the outer side of the finger he now enters with a drilling motion the trocar and canula, till he feels the point strike the horizontal portion of the pubis just to the inner side of the spine of that bone. The point is then to be slightly retracted and turned upwards or downwards; the instrument is then to be further introduced till the point moves freely in all directions, showing it to be fairly lodged in the cavity of the sac. The point of the instrument should now be turned into the inguinal canal, for the purpose of scarifying freely the inner surface of the upper part of the sac, as well as that just below the internal ring. The trocar is now to be withdrawn, and the surgeon, again ascertaining that the canula has not been displaced from the cavity of the sac, throws in slowly and cautiously with the syringe, which should be held nearly vertical, half a drachm of Lugol's solution of iodine, or half a drachm of the tincture of cantharides, which should be lodged as nearly as may be at the orifice of the external ring. The canula is now to be removed, and the operation is completed. A compress should be laid above the upper margin of the external ring, pressed down firmly with the finger, and the truss slid down upon it. The patient is to be kept from changing his position during the application of the truss, and should be confined for a week or ten days to his bed, with his thighs and thorax flexed, keeping up steadily as much pressure with the truss as can be borne without increasing the pain, in order to prevent the viscera from descending and breaking up the new adhesions while they are yet in the forming state, or avoiding the risk of their becoming strangulated or being rendered irreducible by the lymph effused into the cavity of the sac.

"The author has practised this operation in thirteen different cases, in but one of which was there any peritoneal soreness developed that excited the slightest apprehension, and in this case it subsided under the application of leeches and fomentations. In several of these cases a single operation appeared to be perfectly successful. In others—where the sac was larger, or the patient was less careful in keeping the truss steadily applied during the first week, or from a cautiousness in introducing in the first cases a more limited amount of fluid—the effect was merely to narrow the sac, rendering a repetition of the process necessary for the cure. Of the permanency of the cure, during *several years after the operation*, the author is unable to speak, most of the patients operated on being temporary residents of the Philadelphia Hospital, and passing after a few months beyond the reach of enquiry. While under the cognisance of the author, they were employed without a truss as labourers on the farm attached to the institution, and in no one of the cases, during this period, had the hernial tumour recurred. It would, however, be but a proper measure of precaution to direct the truss to be worn subsequently for several months, in order to confirm the cure.

"The greater number of these operations were performed by the author eight years ago, before classes of students at the Philadelphia Hospital, but as he was able to trace the future history of the cases but for a few months only, they were not deemed of sufficient importance for publication. Very recently M. Velpeau has published a process almost precisely the same as that just described." (p. 283.)

Judging from this work, the Americans have had comparatively little experience in lithotrity, nor is there anything of interest in the section on lithotomy. Our author seems to have had little personal experience in either proceeding, and quotes from the works of Messrs. Syme and Fergusson as leading British authorities on those subjects. On lithotomy we are told that,

"This instrument [the gorget], as modified by the late Dr. Physick, in rendering the blade shifting so that it may be separately sharpened and made to bear the keenest edge, is the one generally employed in this country for the division of the prostate. It has probably been employed in two thirds of all the cutting operations for stone done in this city for the last thirty or forty years, and which, as shown by the statistical reports of the Pennsylvania Hospital, have been attended by as large an average of success as those by any other mode of operation. It is the favourite instrument of Professor Dudley, of the Transylvania University, who has operated a greater number of times than any other American surgeon, and with a success that has been unexampled." (p. 329.)

We have seen some very queer-looking American lithotomy instruments, which have not greatly exalted our views of American lithotomy, or American cutlery. Our Greens, our Listons and our Keys ; our Weisses, our Laundys, and our Simpsons may look aghast when they read this :

"It has been objected to the gorget, that it makes the incision too mechanically and *too blindly*, it having no guide for its direction but the groove in the staff, that if it slide from the latter instrument it may plunge between the bladder and the rectum, and that the cutting edge of the gorget, even when it keeps the proper direction, may enter so far as to wound the posterior surface of the bladder. These objections, which might have been tenable against the imperfectly-sharpened instrument heretofore employed in Great Britain, are wholly inapplicable to the keenly-set gorget of Physick, which requires but a gentle effort for its introduction, and in the hands of no one who understands the use of cutting instruments can possibly either slip from the staff or wound the posterior wall of the bladder." (pp. 329-30.)

But we must bring this article to a close. Our disappointment with Dr. Pancoast's production is, we doubt not, sufficiently manifest. We were led to expect something totally different. We expected in reality to see 'Pancoast's Operative Surgery,' when we read this title on the one side of the volume, and were not prepared to find that we had before us little else than a portion of Bourgery's work on the same subject. Though we are carefully told in the "advertisement," that Bourgery is "but little more than half completed," it has sufficed to fill our author's volume, which comprises, to use his own language, "the operative surgery of the day."

We refrain from giving a deliberate opinion upon Bourgery at the present time, but have no hesitation in saying that the operative surgery of that work is more an exposition of French surgery than is entirely to our taste. If our "author" and publishers together, chose to disseminate this style of surgery, or if the Americans are pleased with it, we have no reason to object, but we must protest against this method of bringing the subject forward. We think it hard enough upon real authors and publishers who have been at great labour and expense upon original works, to have their property seized upon, as it often is, and sent forth to the American public, with the addition of an American editor's name; but this book shows a new feature in "the trade." Here is the unfortunate Doctor Bourgery so squeezed, so mutilated, so truly "translated," (in Bottom's phrase,) that for a time he will scarcely be able to recognize himself; authors knowing well that they cannot have any of the emoluments from these American reprints, may surely be permitted the gratification of seeing their names appended to their own works, even though placed in juxta-position with those of American editors, who have made the productions "complete" with some notes and amendments. But it is too bad that appropriation should be carried so far as to strike an author's name from a title-page, and substitute that of a commentator. Had this work appeared as a translation or abridgment of a portion of Bourgery with alterations, notes, and additions by Dr. Pancoast, we should not have complained, and should possibly have given Dr. Pancoast credit for his annotations; but we can scarcely find terms sufficiently strong wherewith to express our opinion of this novel method of appropriating copy-right.

In our opinion Dr. Pancoast would have produced a much more valuable volume had he given more from himself and less from Bourgery, and the whole might have been published in a less costly and more portable style. As a work of reference it is much inferior to the original Bourgery, and infinitely behind the admirable treatise of Velpeau; and as a handbook for the practitioner, it is not to compare with those which have issued from our own press within the last eight or ten years; works which we believe are in extensive circulation among our author's countrymen, in the shape of American editions.

We do not think it necessary to criticise our author's style, although it is anything but unexceptionable, but we have great fault to find with the references to the plates and figures. Some of the plates are not numbered at all, and the errors of reference as regard the figures are of frequent occurrence, and sorely trying to the patience of the reader. In our copy we have searched in vain for plate 28, (possibly the binder's fault,) which exhibits the author's mode of excising the elbow.

---

